# Diet, body size and menarche in a multiethnic cohort

**DOI:** 10.1038/sj.bjc.6690303

**Published:** 1999-04

**Authors:** C Koprowski, R K Ross, W J Mack, B E Henderson, L Bernstein

**Affiliations:** Department of Preventive Medicine, School of Medicine, University of Southern California, Los Angeles, CA, 90033, USA

**Keywords:** menarche, diet, height, body mass index

## Abstract

A multiethnic cohort of 1378 Southern California school girls aged 8–13 years was followed for 4 years to evaluate factors predicting age at menarche, a risk factor for breast cancer. Height and weight were measured and dietary intake was assessed using a semi-quantitative food frequency questionnaire. Of 939 girls providing data on menarcheal status, 767 were premenarcheal at the start of the study; 679 girls provided acceptable dietary data and were included in the analyses. Cox proportional hazards models were used to assess the relationship between diet, body size, ethnicity and age at menarche. Hispanic, Asian/Pacific Island and African-American girls were more likely to experience early menarche than non-Hispanic white girls. Tall (> 148.6 cm) versus short (< 135.9 cm) girls experienced earlier menarche (relative hazard (RH) = 2.9, 95% confidence interval (CI) 2.1–4.1) as did those with high Quetelet's index (QI, kg m^−2^) (> 20.7) versus low QI (< 16.1) (RH = 2.2, 95% CI 1.7–2.9). Of all the dietary variables analysed, only energy intake was related to age at menarche. High versus low energy intake (> 12013 kJ vs < 7004 kJ) was associated with a delay in menarche (RH = 0.7, 95% CI 0.5–0.9); this finding was limited to a subset of heavy Hispanic girls who appeared to underreport their dietary intake. © 1999 Cancer Research Campaign


*British Journal of Cancer* (1999) **79**(11/12), 1907–1911
© 1999 Cancer Research Campaign
Article no. bjoc.1998.0303

### Diet, body size and menarche in a multiethnic cohort

**C Koprowski, RK Ross, WJ Mack, BE Henderson and L Bernstein**

Department of Preventive Medicine, School of Medicine, University of Southern California, Los Angeles, CA 90033, USA

**Summary** A multiethnic cohort of 1378 Southern California school girls aged 8–13 years was followed for 4 years to evaluate factors predicting age at menarche, a risk factor for breast cancer. Height and weight were measured and dietary intake was assessed using a semi-quantitative food frequency questionnaire. Of 939 girls providing data on menarcheal status, 767 were premenarcheal at the start of the study; 679 girls provided acceptable dietary data and were included in the analyses. Cox proportional hazards models were used to assess the relationship between diet, body size, ethnicity and age at menarche. Hispanic, Asian/Pacific Island and African-American girls were more likely to experience early menarche than non-Hispanic white girls. Tall (> 148.6 cm) versus short (< 135.9 cm) girls experienced earlier menarche (relative hazard (RH) = 2.9, 95% confidence interval (CI) 2.1–4.1) as did those with high Quetelet's index (QI, kg $m^{-2}$) (> 20.7) versus low QI (< 16.1) (RH = 2.2, 95% CI 1.7–2.9). Of all the dietary variables analysed, only energy intake was related to age at menarche. High versus low energy intake (> 12 013 kJ vs < 7004 kJ) was associated with a delay in menarche (RH = 0.7, 95% CI 0.5–0.9); this finding was limited to a subset of heavy Hispanic girls who appeared to underreport their dietary intake.

**Keywords:** menarche; diet; height; body mass index

A number of risk factors for breast cancer have been identified. Late age at first full-term pregnancy, nulliparity, early age at menarche and late age at menopause are factors considered to increase the risk of breast cancer (Willett, 1989; Kelsey and Bernstein, 1996). The association between these reproductive risk factors and breast cancer suggests that female sex hormones play a role in the aetiology of this disease (MacMahon et al, 1973; Pike, 1987). Up to 50% of the difference in incidence rates between high- and low-risk countries (e.g. USA and Japan) can be explained by corresponding differences in age at menarche and post-menopausal weight (Hoel et al, 1983). A 1-year decrease in age at menarche is estimated to increase breast cancer risk by at least 10% (Ursin et al, 1994).

A woman's age at her first menstruation may be determined in part by such factors as nutrition, body composition, genetics, altitude of residence, sleep patterns, family size and health status (Warren, 1990; Golub, 1992; Murata and Araki, 1993). Over the past century, the average age at menarche has declined in most population groups studied (Hoel et al, 1983; Wyshak, 1983; Warren, 1990). The rate of decline has decreased in some populations in recent decades (Wyshak, 1983; Wellens et al, 1990; Rees, 1993; Tryggvadottir et al, 1994). Since this downward trend in age at menarche has occurred to a greater extent in developed than in developing countries, it has been attributed to improved nutrition and socioeconomic conditions (Meyer et al, 1990).

Although nutritional status may be important, no specific relationship between diet and age at menarche has been clearly established. Among those studies that have controlled for other factors associated with onset of menarche, some have found an association between higher total energy intake and earlier age at menarche (Meyer et al, 1990; Moisan et al, 1990*b*), whereas others have not (Moisan et al, 1990*a*; Maclure et al, 1991; Merzenich et al, 1993). Studies that have looked at specific components of diet are similarly inconclusive (Meyer et al, 1990; Moisan et al, 1990*a*; Maclure et al, 1991). Differences in study design and methodology make comparisons of studies difficult. For example, measurement of dietary intake has varied from observation of children's eating habits (Hill et al, 1980) to a combination of food records and food frequency questionnaires (Merzenich et al, 1993).

In 1988, we established a multiethnic cohort of schoolgirls in Southern California to evaluate whether certain aspects of diet predicted age at menarche. Understanding factors that affect onset of menarche should lead to a better understanding of the aetiology of breast cancer.

### MATERIALS AND METHODS

We recruited 1386 4th to 7th grade girls who were between the ages of 8 and 13 years and who were attending one of 14 Catholic schools in the San Gabriel Valley area of Los Angeles County, California in 1988. Parents were sent letters explaining the purpose of the study and allowing them to decline their daughter's participation in the cohort study. The parents of two subjects refused permission to participate, while six students agreed to participate but never completed an assessment. We collected data from a cohort of 1378 girls, including 397 (28.8%) non-Hispanic white, 771 (56.0%) Hispanic white, 164 (11.9%) Asian/Pacific Island and 46 (3.3%) African-American students. Two assessments of diet were performed during the first year (approximately 6 months apart) and assessments were conducted annually for the remaining 3 years of the study. Each girl participated in one to five assessments during the course of the study. This report uses data collected at each subject's first assessment.

We obtained reports on age at menarche at assessments four and five, and from questionnaires completed annually by parents. We

*Received 16 June 1998*
*Revised 2 September 1998*
*Accepted 7 September 1998*

*Correspondence to:* C Koprowski, USC/Norris Comprehensive Cancer Center, 1441 Eastlake Ave., MS44, Room 3422, Los Angeles, CA 90033, USA

**Table 1** Means (SD) of selected variables by ethnic group and for all subjects combined

| | Ethnic group | | | | | |
|---|---|---|---|---|---|---|
| | **Non-Hispanic white *n* = 218** | **Hispanic white *n* = 356** | **Asian/Pacific Island *n* = 80** | **African-American *n* = 25** | ***P*-value[a]** | **All subjects *n* = 679** |
| Age at first assessment | 10.5 (1.1) | 10.7 (1.0) | 10.4 (0.9) | 10.8 (1.2) | 0.06 | 10.6 (1.0) |
| Weight (kg) | 36.7 (9.3)* | 39.9 (10.2)† | 34.6 (10.2)* | 42.5 (11.4)† | 0.001 | 38.3 (10.1) |
| Height (cm) | 142.2 (8.9)* | 142.6 (8.8)* | 139.7 (9.0)* | 148.1 (10.4)† | 0.001 | 142.3 (9.0) |
| QI[b] | 18.1 (3.3)* | 19.5 (3.8)† | 17.5 (3.2)* | 19.1 (4.2)*† | 0.001 | 18.8 (3.7) |
| Energy (kJ)[c] | 9510 (3420) | 10 000 (4000) | 9600 (3770) | 10 670 (3540) | 0.29 | 9820 (3780) |
| Carbohydrate (g) | 302 (118) | 322 (139) | 313 (127) | 336 (127) | 0.28 | 315 (131) |
| Protein (g) | 89 (34) | 91 (40) | 91 (39) | 98 (32) | 0.70 | 90 (38) |
| Fat (g) | 81 (31) | 85 (37) | 78 (35) | 93 (32) | 0.12 | 83 (35) |

[a]ANOVA *P*-value. Bonferoni adjustments of *P*-values were used for multiple pairwise comparisons. Means with superscripts in common (denoted by * and †) did not differ statistically. [b]QI = Quetelet's index, kg $m^{-2}$. [c]kilocalorie (kcal) = 4.184 kilojoule (kJ); all nutrients are reported (unadjusted) intakes.

**Table 2** Adjusted relative hazards (RH) for menarche by ethnic group, with 95% confidence intervals (95% CI)[a]

| **Ethnic group** | **% Achieving menarche** | **Median age at menarche (years)** | **RH (95% CI)** |
|---|---|---|---|
| Non-Hispanic white | 58.7 (128/218) | 12.8 | 1.0 |
| Hispanic white | 73.3 (261/356) | 12.4 | 1.6 (1.2–1.9) |
| Asian/Pacific Island | 72.5 (58/80) | 12.2 | 2.1 (1.5–2.9) |
| African-American | 68.0 (17/25) | 12.1 | 1.8 (1.0–2.9) |

[a]Cox proportional hazards model adjusted for age at first assessment, height and Quetelet's index (kg $m^{-2}$).

obtained information on whether or not subjects experienced menarche for 939 of the 1378 students. Of these 939 girls, 767 girls either began menstruating during the study (following their first assessment) or were premenarcheal at the time of last contact. This report is limited to 758 premenarcheal girls who completed dietary assessments and were weighed and measured for height at their first assessment.

Dietary data were collected using a 7-day food frequency questionnaire (FFQ) based on the Semiquantitative Food-Frequency Questionnaire used in the Nurses' Health Study (Willett et al, 1985) and adapted for use with adolescents. The adapted FFQ used in this study contained the same list of food items, but excluded alcohol, coffee and tea. The original FFQ asked subjects to recall dietary intake during the previous year; however, for our study subjects were asked to recall how often each item was consumed during the previous week. We adapted the FFQ to this time frame based on Frank's report (Frank, 1994) that most children in this age range (8–13 years old initially) are able to recall dietary intake for the previous week, but may have conceptual difficulties recalling dietary intake for the previous year. The eight response categories for frequency of consumption of a food item ranged from 'Never' to '6 or more times per day'. Students were asked to indicate the amount of sugar added to food as well as the brands of cereals eaten during the previous 7 days. Students were also asked to write down foods consumed during the previous 7 days that were not included among the 110 food items listed. Students completed the FFQ in a classroom setting with a supervisor leading students through the assessment and an assistant circulating through the classroom to answer questions. Food models were used to help students with portion sizes. The Channing Laboratory, Harvard University, provided nutrient analyses.

Height and weight were measured annually. Students were weighed without shoes or oversweaters using a digital electronic scale. The scale was calibrated daily against a weight balance scale to assure accuracy. Height was measured using a rigid measuring stick, calibrated in inches, that was taped to the wall. Each student was asked to face forward with the back of her head against the stick. A ruler was then placed on top of the subject's head and the height was read from the measuring stick. Two people performed the measurements: the supervisor doing the measuring and the assistant recording data. Height and weight were used to calculate Quetelet's index (QI, weight(kg)/height(m)$^2$) as a measure of weight corrected for height.

To determine the plausibility of dietary data, we identified energy intake outliers using the Schofield formulae for basal metabolic rate (BMR) (Schofield, 1985). These equations provide the best estimate for resting energy expenditure in children (Firouzbakhsh et al, 1993). The Schofield formulae for young females are as follows:

1. for age 3.0–9.9 years: BMR (MJ per day) = 0.071 weight(kg) + 0.677 height(m) + 1.553
2. for age 10.0–16.0 years: BMR (MJ per day) = 0.035 weight(kg) + 1.948 height(m) + 0.837

Subjects were removed from the analysis if their reported energy intake from the FFQ was less than 80% of or greater than four times their estimated BMR (79/758, or 10.4% of subjects were removed, leaving a total of 679 subjects). Merzenich et al (1993) used energy intake less than BMR as a lower exclusion criterion for their study, but did not specify whether an upper exclusion criterion was used. No specific cut-off points for energy intake relative to BMR have been established to define acceptable and plausible energy intake values.

 *© Cancer Research Campaign 1999*

**Table 3** Relative hazards (RH) for menarche associated with measures of height and QI[a] at first assessment, with 95% confidence intervals (95% CI) (based on 679 subjects)

| | % Achieving menarche | Model 1[b] RH (95% CI) | Model 2[c] RH (95% CI) |
|---|---|---|---|
| Height (cm) | | | |
| < 135.9 | 45.4 (74/163) | 1.0 | 1.0 |
| 135.9–142.2 | 67.2 (107/158) | 1.6 (1.2–2.1) | 1.5 (1.1–2.1) |
| 142.2–148.6 | 75.3 (134/178) | 2.0 (1.5–2.8) | 1.8 (1.3–2.5) |
| > 148.6 | 82.8 (149/180) | 2.9 (2.1–4.1) | 2.6 (1.8–3.7) |
| *P*-value[d] | | < 0.001 | < 0.001 |
| QI | | | |
| < 16.1 | 54.3 (89/164) | 1.0 | 1.0 |
| 16.1–18.0 | 65.9 (114/173) | 1.6 (1.2–2.1) | 1.5 (1.1–2.0) |
| 18.0–20.7 | 73.8 (124/168) | 1.9 (1.5–2.6) | 1.8 (1.3–2.4) |
| > 20.7 | 78.7 (137/174) | 2.2 (1.7–2.9) | 1.7 (1.3–2.3) |
| *P*-value | | < 0.001 | 0.001 |

[a]QI = Quetelet's index, kg $m^{-2}$. [b]Model includes age at first assessment. [c]Model includes age at first assessment, ethnicity, QI and height. [d]Test for trend.

**Table 4** Adjusted relative hazards (RH) for menarche associated with total energy intake and adjusted nutrients, with 95% confidence intervals (95% CI) (based on 679 subjects)

| Nutrient | Model 1[a] RH (95% CI) | Model 2[a,b] RH (95% CI) |
|---|---|---|
| Energy (kJ) | | |
| < 7004 | 1.0 | 1.0 |
| 7004–9152 | 0.9 (0.7–1.1) | 0.9 (0.7–1.1) |
| 9152–12013 | 0.7 (0.5–0.9) | 0.7 (0.5–0.9) |
| > 12013 | 0.7 (0.6–0.9) | 0.7 (0.5–0.9) |
| *P*-value[c] | 0.004 | <0.001 |
| Carbohydrate (g) | | |
| < 270.4 | 1.0 | 1.0 |
| 270.4–290.9 | 0.9 (0.7–1.2) | 0.9 (0.7–1.2) |
| 290.9–313.6 | 1.1 (0.8–1.4) | 1.1 (0.8–1.4) |
| > 313.6 | 1.0 (0.8–1.3) | 0.9 (0.7–1.2) |
| *P*-value | 0.78 | 0.90 |
| Protein (g) | | |
| < 73.7 | 1.0 | 1.0 |
| 73.7–84.4 | 1.2 (0.9–1.5) | 1.1 (0.8–1.4) |
| 84.4–95.3 | 1.1 (0.9–1.5) | 1.2 (0.9–1.5) |
| > 95.3 | 1.1 (0.8–1.5) | 1.1 (0.8–1.5) |
| *P*-value | 0.51 | 0.36 |
| Fat (g) | | |
| < 69.8 | 1.0 | 1.0 |
| 69.8–77.0 | 1.2 (0.9–1.6) | 1.3 (1.0–1.6) |
| 77.0–85.0 | 0.9 (0.7–1.2) | 1.0 (0.7–1.3) |
| > 85.0 | 0.9 (0.7–1.2) | 1.0 (0.8–1.3) |
| *P*-value | 0.28 | 0.56 |

[a]Both models include age at first assessment, height and QI (Quetelet's index, kg/$m^{-2}$). Carbohydrate, protein and fat adjusted by linear regression of the log of nutrient intake on continuous variables for height and QI and the log of energy intake. Energy intake adjusted for height and QI. [b]Model 2 also includes ethnicity and energy intake, with the exception of the energy intake model, which includes only ethnicity. [c]Test for trend.

The final study population of girls consisted of 218 non-Hispanic whites, 356 Hispanic whites, 80 Asians/Pacific Islanders and 25 African-Americans. The final study population was not significantly different in terms of ethnicity or age from subjects who did not meet these final criteria ($P > 0.05$).

Dietary nutrients were adjusted for energy intake, height and QI according to the method of Willett and Stampfer to remove the potential confounding effects of energy intake and body size (Willett and Stampfer, 1986). Nutrient intakes (carbohydrate, protein and fat) were adjusted by regressing the log of each nutrient on the log of total energy intake, height and QI. Total energy intake was adjusted by the linear regression of the log of energy intake on continuous variables for height and QI. The residuals from each of these regressions were used in all analyses, with the overall mean intake of the nutrient added to each residual to increase interpretability (Willett and Stampfer, 1986). The term 'adjusted nutrients' is used when referring to nutrients adjusted by this method.

We assessed the relationship between energy intake, adjusted nutrients, height and QI and the age at onset of menses using the Cox proportional hazards model (Lee, 1992). We refer to the estimated hazard ratio obtained from the Cox proportional hazards model as the relative hazard (RH). Analyses were conducted using the Epicure software package, which allows for left-truncated (or staggered) entry time (Preston et al, 1993). Left-truncated entry times were important in these analyses since the entry time for each subject was age when first assessed (which varied from 8 to 13 years). Exit time was age at menarche or last contact for those not achieving menarche during the study period with menstrual status used as the event indicator. We included age at assessment in all models.

Body size (height and QI) and adjusted nutrient variables (energy, carbohydrate, fat and protein) were categorized into quartiles based on the distribution of the entire cohort. We used the likelihood ratio test to test for overall linear trend in the RH across categories and the Wald method to calculate 95% confidence intervals (95% CI) for RHs.

We compared means of selected variables between ethnic groups with analysis of variance methods using Bonferoni adjustments of the *P*-values for pairwise comparisons (SAS Institute Inc., 1997).

### RESULTS

Subjects, who ranged in age from 8 to 13 years, were in the 4th through 7th grades at the time of initial assessment. Of these subjects, 464 (68.3%) began menstruating during the course of the study while 215 (31.7%) were premenarcheal at the time of last contact. Ages at menarche ranged from 9.7 to 14.8 years.

We did not observe significant differences in mean age at first assessment between the different ethnic groups (Table 1). On average, Asian/Pacific Island girls weighed less than girls from the other ethnic groups, were shorter and had lower body mass as measured by QI. In contrast, African-American girls were taller and weighed more, while Hispanic girls had a higher QI. Mean intake of energy, protein and carbohydrate did not significantly differ among ethnic groups ($P > 0.05$).

The median age at menarche for all subjects combined was 12.5 years, ranging from 12.8 years for non-Hispanic whites to 12.1 years for African-Americans (Table 2). We observed ethnicity to be a significant predictor of menarche after adjustments were made for age at assessment, height and QI. Asian/Pacific Island girls experienced menarche earlier than non-Hispanic white girls (RH = 2.1, 95% CI 1.5–2.9) as did Hispanic and African-American girls (RH = 1.6 and 1.8 respectively).

Both QI and height were significantly related to the age at onset of menarche (Table 3). The tallest girls (height > 148.6 cm) at the time of first assessment reached menarche at an earlier age than

© Cancer Research Campaign 1999

**Table 5** Multivariate relative hazards (RH) and 95% confidence intervals (95% CI) for menarche associated with reported energy intake quartile by ethnicity. Model adjusted for age at assessment, height and QI[a]

| Energy intake quartile | RH (95% CI) | | | |
|---|---|---|---|---|
| | Hispanics | | non-Hispanics | |
| | Hispanics ($n$ = 356) | Excluding 'heavy' Hispanics[b] ($n$ = 265) | All ($n$ = 323) | Whites ($n$ = 218) |
| 1 | 1.0 | 1.0 | 1.0 | 1.0 |
| 2 | 0.7 (0.5–0.9) | 0.8 (0.5–1.2) | 1.0 (0.7–1.5) | 1.1 (0.6–1.7) |
| 3 | 0.6 (0.4–0.8) | 0.8 (0.5–1.2) | 0.7 (0.5–1.1) | 0.9 (0.6–1.5) |
| 4 | 0.5 (0.4–0.8) | 0.7 (0.4–1.0) | 1.0 (0.7–1.5) | 1.0 (0.6–1.6) |
| *P*-value[c] | 0.0005 | 0.08 | 0.55 | 0.73 |

[a]Quartiles based on each ethnic group distribution, QI = Quetelet's index, kg $^{-2}$. [b]Analysis restricted to Hispanic girls in the three lowest QI quartiles. [c]Test for trend.

the shortest girls (height < 135.9 cm); the RH was 2.9 (95% CI 2.1–4.1). Similarly, we observed that girls with the largest body mass (QI > 20.7) reached menarche sooner than girls with the smallest body mass (QI < 16.1, RH = 2.2, 95% CI 1.7–2.9). Height and QI were independently related to age at menarche, even after adjustment for ethnicity. We found no evidence for an interaction between height and QI (data not shown). The interaction between body size and ethnicity was examined among non-Hispanic white, and Hispanic whites as there were too few Asian/Pacific Islander and African-American subjects for this assessment. Within Hispanic and non-Hispanic whites, we found no evidence of an interaction between ethnicity and body size (data not shown).

We tested the assumption that QI is a measure of body mass that is uncorrelated with height by calculating the correlation coefficient between height and QI. A statistically significant correlation of 0.33 ($P$ = 0.0001) was observed between QI and height.

We examined the relationship between diet and age at menarche using a series of models that adjusted for various combinations of factors (Table 4). Total energy intake was the only nutrient to show evidence of a dose–response relationship; high total energy intake was associated with a delay in menarche. The RH comparing the highest energy intake quartile (> 12 013 kJ) to the lowest (< 7004 kJ) was 0.7 after adjusting for age at assessment, height, QI and ethnicity ($P_{trend}$ < 0.001). Carbohydrate, protein and fat intake (adjusted for age at first assessment, height and QI) were unrelated to age at menarche in any model (Table 4).

Separate analyses were done for Hispanics, all non-Hispanics, and non-Hispanic whites. The dose–response relationship between energy intake and menarche was limited to Hispanic whites (Table 5). When Hispanic girls from the highest QI category were excluded from the analysis (assuming they might be under-reporting their total energy intake), the inverse relationship between energy intake and risk of earlier menarche was still apparent, but energy intake was not a significant predictor of menarche in this ethnic group ($P$ = 0.08).

### DISCUSSION

This study has demonstrated that measures of body size are associated with age at menarche. The relationships between body size (height and QI) and menarche are consistent with those observed in other studies (Moisan et al, 1990*a*, 1990*b*; Maclure et al, 1991; Merzenich et al, 1993). A number of investigators have proposed that age at menarche is closely related to skeletal maturity (Ellison, 1982; Elizondo, 1992). The findings from this study demonstrate that skeletal development, as measured by height, is related to menarche. Holding age at initial assessment constant, taller girls were more likely to experience menarche at an earlier age. The effect of height was independent of the effects of QI and ethnicity on age at menarche.

The relationship between body size and menarche is often assessed using body mass index (QI) as a measure of body fat. An association between menarche and QI has been observed in several studies (Meyer et al, 1990; Maclure et al, 1991; Merzenich et al, 1993). QI is assumed to be a measure of weight uncorrelated with height and, as such, to be proportional to the amount of body fat (i.e. a higher body mass index is indicative of a larger amount of body fat), but the validity of this assumption is untested in premenarcheal girls. In our study, a statistically significant correlation was observed between QI and height for premenarcheal girls suggesting that, prior to menarche, QI may not be proportional to the amount of body fat and thus may not be the optima measure. We evaluated other measures of body mass, such as weight divided by height or the square root of height, but these also were correlated with height.

The role of ethnicity in the timing of menarche within a population has not been well studied. Our results demonstrate ethnic differences in age at menarche for a Southern California population of girls. Hispanic, Asian/Pacific Island and African-American girls achieved menarche earlier than non-Hispanic white girls. After Asian women immigrate to the USA, breast cancer risk is observed to increase over several generations, nearing that of US whites (Ziegler et al, 1993; Shimizu et al, 1991). A large proportion of Asian/Pacific Island girls in our study were Filipinas. Based on our results, earlier age at menarche may, in part, explain the changing breast cancer risk observed for Asian-Americans and the greater risk of Filipino women relative to other Asian-Americans (Bernstein et al, 1995).

Studies of adequately nourished subjects have been unable to show any clear relationship between diet and age at menarche (Meyer et al, 1990; Moisan et al, 1990*a*, 1990*b*; Maclure et al, 1991; Merzenich et al, 1993); however, girls who are malnourished or inadequately nourished seem to experience menarche at later ages than well nourished girls (Warren, 1990; Frisch, 1994). We found no evidence to support an independent role of dietary macronutrients in determining menarche when diet was measured

© Cancer Research Campaign 1999

as carbohydrate, protein and fat intake. However, our results suggested that higher energy intake was associated with a delay in menarche. These results are contradictory to what we initially hypothesized. When separate analyses were done by ethnic group (Hispanics vs non-Hispanics), the negative dose–response relationship between energy intake and menarche was observed only among Hispanic girls. Under-reporting of dietary intake by overweight or obese subjects has been documented previously (Black et al, 1993; Maffeis et al, 1994; Klesges et al, 1995; Ballard-Barbash et al, 1996). In an effort to determine if Hispanic girls with high QI were under-reporting their dietary intake, we repeated the analyses excluding girls from the highest QI category. The reduced strength of the relationship between energy intake and menarche observed when Hispanic girls from the highest QI category were removed suggests that part of the negative relationship between energy intake and menarche observed in the entire Hispanic population may have been due to under-reporting of dietary intake among girls with the highest QI.

The timing of dietary measurements in relation to the onset of first menses may have negatively influenced our results as well as those of other investigators (Meyer et al, 1990; Moisan et al, 1990*a*, 1990*b*; Maclure et al, 1991; Merzenich et al, 1993). The time lag between our initial assessment of dietary intake and menarche ranged from less than 1 week to 3.2 years prior to menarche. It may be that diet during early childhood, diet 6 months prior to menarche or diet during some other time frame is the more critical element to measure.

While the results of this study do not indicate any relationship between dietary nutrients per se and menarche, they further support that body size, independent of chronological age, is a predictor of age at menarche. Although body size as measured by QI is related to age at menarche, it remains correlated with height among premenarcheal girls. Since ethnic differences in age at menarche were observed in a population that is relatively uniform in terms of socioeconomic factors; hereditary factors may also influence age at menarche.

### ACKNOWLEDGEMENTS

Supported by grant ACS-3-7-95 from the American Cancer Society, California Division, Inc. and the L.K. Whittier Foundation, a private foundation supporting biomedical research.

### REFERENCES

Ballard-Barbash R, Graubard I, Krebs-Smith SM, Schatzkin A and Thompson FE (1996) Contribution of dieting to the inverse association between energy intake and body mass index. *Eur J Clin Nutr* **50**: 98–106

Bernstein L, Miu A, Monroe K, Henderson BE and Ross RK (1995) Cancer incidence among Filipinos in Los Angeles County, 1972–1991. *Int J Cancer* **63**: 345–348

Black AE, Prentice AM, Goldberg GR, Jebb SA, Bingham SA, Livingstone MBE and Coward WA (1993) Measurements of total energy expenditure provide insights into the validity of dietary measurements of energy intake. *J Am Diet Assoc* **93**: 572–579

Elizondo S (1992) Age at menarche: its relation to linear and ponderal growth. *Ann Hum Biol* **19**: 197–199

Ellison PT (1982) Skeletal growth, fatness and menarcheal age: a comparison of two hypotheses. *Hum Biol* **54**: 269–281

Firouzbakhsh S, Mathis RK, Dorchester WL, Oseas RS, Groncy PK, Grant KE and Finklestein JZ (1993) Measured resting energy expenditure in children. *J Pediatric Gastroenterol Nutr* **16**: 136–142

Frank GC (1994) Environmental influences on methods used to collect dietary data from children. *Am J Clin Nutr* **59**(suppl): 207s–211s

Frisch RE (1994) The right weight: body fat, menarche and fertility. *Proc Nutr Soc* **53**: 113–129

Golub S (1992) Menarche: the onset of menstruation. In *Periods: From Menarche to Menopause*, pp. 24–51. Sage Publications: Newbury Park

Hill P, Wynder EL, Garbaczewski P, Hellman P and Hill M (1980) Diet and menarche in different ethnic groups. *Eur J Cancer* **16**: 519–525

Hoel DG, Wakabayashi T and Pike MC (1983) Secular trends in the distributions of the breast cancer risk factors – menarche, first birth, menopause and weight – in Hiroshima and Nagasaki, Japan. *Am J Epidemiol* **118**: 78–89

Kelsey JL and Bernstein L (1996) Epidemiology and prevention of breast cancer. *Annu Rev Public Health* **17**: 47–67

Klesges RC, Eck LH and Ray JW (1995) Who underreports dietary intake in a dietary recall? Evidence from the Second National Health and Nutrition Examination Survey. *J Consult Clin Psychol* **63**: 438–444

Lee ET (1992) Identification of prognostic factors related to survival time. In *Statistical Methods for Survival Data Analysis*, pp. 243–280. John Wiley: New York

Maclure M, Travis LB, Willett WC and MacMahon B (1991) A prospective cohort study of nutrient intake and age at menarche. *Am J Clin Nutr* **54**: 649–656

MacMahon B, Cole P and Brown J (1973) Etiology of human breast cancer: a review. *J Natl Cancer Inst* **50**: 21–42

Maffeis C, Schutz Y, Zaffanello M, Piccoli R and Pinelli L (1994) Elevated energy expenditure and reduced energy intake in obese prepubertal children: paradox of poor dietary reliability in obesity? *J Pediatr* **124**: 348–354

Merzenich H, Boeing H and Wahrendorf J (1993) Dietary fat and sports activity as determinants for age at menarche. *Am J Epidemiol* **138**: 217–224

Meyer F, Moisan J, Marcoux D and Bouchard C (1990) Dietary and physical determinants of menarche. *Epidemiology* **1**: 377–381

Moisan J, Meyer F and Gingras S (1990*a*) Diet and age at menarche. *Cancer Causes Control* **1**: 149–154

Moisan J, Meyer F and Gingras S (1990*b*) A nested case-control study of the correlates of early menarche. *Am J Epidemiol* **132**: 953–961

Murata K and Araki S (1993) Menarche and sleep among Japanese schoolgirls: an epidemiological approach to onset of menarche. *Tohoku J Exp Med* **141**: 21–27

Pike MC (1987) Endogenous hormones. In *Cancer*: *Risks and Prevention,* Vessey, MP and Gray M (eds), pp. 195–210. Oxford University Press: Oxford

Preston D, Lubin J, Pierce D and McConney M (1993) *Epicure*. HiroSoft International: Seattle

Rees M (1993) Menarche when and why [commentary]. *Lancet* **342**: 1375–1376

SAS Institute Inc. (1997) *Statistical Applications Software*. SAS Institute Inc.: Cary, NC

Schofield C (1985) Predicting basal metabolic rate, new standards and review of previous work. *Human Nutrition*: *Clinical Nutrition* **39c**(suppl 1): 5–42

Shimizu H, Ross RK, Bernstein L, Yatani R, Henderson BE and Mack TM (1991) Cancers of the prostate and breast among Japanese and white immigrants in Los Angeles County. *Br J Cancer* **63**: 936–966

Tryggvadottir L, Tulinius H and Larusdottir M (1994) A decline and a halt in mean age at menarche in Iceland. *Ann Hum Biol* **21**: 179–186

Ursin G, Bernstein L and Pike MC (1994) Breast cancer. In *Trends in Cancer Incidence and Mortality*, Vol. 19, Doll R, Fraumeni JF Jr and Muir CS (eds), pp. 241–264. Cancer Surveys: Advances and Prospects in Clinical, Epidemiological and Laboratory Oncology. Cold Spring Harbor Laboratory Press: Cold Spring Harbor

Warren MP (1990) Metabolic factors and the onset of puberty. In *Control of the Onset of Puberty*, Grumbach MM, Sizonenko PC and Aubert ML (eds), pp. 553–573. Williams & Wilkins: Baltimore

Wellens R, Malina RM, Beunen G and Lefevre J (1990) Age at menarche in Flemish girls: current status and secular change in the 20th century. *Ann Hum Biol* **14**: 145–152

Willett WC (1989) The search for the causes of breast and colon cancer. *Nature* **338**: 389–394

Willett WC and Stampfer MJ (1986) Total energy intake: implications for epidemiologic analyses. *Am J Epidemiol* **124**: 17–27

Willett WC, Sampson L, Stampfer MJ, Rosner B, Bain C, Witschi J, Hennekens CH and Speizer FE (1985) Reproducibility and validity of a semiquantitative food frequency questionnaire. *Am J Epidemiol* **122**: 51–65

Wyshak G (1983) Secular changes in age at menarche in a sample of US women. *Ann Hum Biol* **10**: 75–77

Ziegler RG, Hoover RN, Pike MC, Hildesheim A, Nomura MY, West DW, Wu-Williams A, Kolonel LN, Horn-Ross PL, Rosenthal JF and Hyer MB (1993) Migration patterns and breast cancer risk in Asian-American women. *J Natl Cancer Inst* **85**: 1819–1827

© Cancer Research Campaign 1999
